# Prevalence of Anxiety, Depression, and Perceived Stress Among Family Caregivers of Patients Diagnosed With Oral Cancer in a Tertiary Care Hospital in Central India: A Cross-Sectional Study

**DOI:** 10.7759/cureus.47100

**Published:** 2023-10-16

**Authors:** Parth Belapurkar, Sourya Acharya, Samarth Shukla, Sunil Kumar, Kashish Khurana, Neema Acharya

**Affiliations:** 1 Department of Medicine, Jawaharlal Nehru Medical College, Datta Meghe Institute of Higher Education and Research (Deemed to be University), Wardha, IND; 2 Department of Pathology, Jawaharlal Nehru Medical College, Datta Meghe Institute of Higher Education and Research (Deemed to be University), Wardha, IND; 3 Department of Obstetrics and Gynaecology, Jawaharlal Nehru Medical College, Datta Meghe Institute of Higher Education and Research (Deemed to be University), Wardha, IND

**Keywords:** prevalence, india, psychosocial factors, perceived stress, depression, anxiety, family caregivers, oral cancer

## Abstract

Background and objectives

Oral cancer is a significant health issue in India, with one of the highest incidence rates globally. Family caregivers play a crucial role in the care of oral cancer patients, but their mental health often faces challenges. This study aimed to assess the prevalence of anxiety, depression, and perceived stress among family caregivers of oral cancer patients in a tertiary care hospital in central India and explore the associated psychosocial factors.

Population and method

The study was carried out between March 2023 and August 2023 in a tertiary care hospital in Wardha, Maharashtra. Family caregivers (N=82, mean age = 36.1 (SD 10.5) years) of patients with clinically diagnosed oral cancer were subjected first to structured psychiatric clinical interviews to screen for psychiatric diagnoses and then were given self-reporting questionnaires for socio-demographic data, Beck Depression Inventory (BDI-II) for measuring the emotional, cognitive, and motivational symptoms of depression, Manifest Anxiety Scale (MAS) to assess the degree of anxiety and Perceived Stress Scale-10 (PSS-10) to assess stress level. Data was analysed using IBM SPSS Statistics for Windows, Version 26.0 (Released 2019; IBM Corp., Armonk, New York, United States). Chi-square test and logistic regression analyses were conducted wherever appropriate in order to explore predictive factors of depressive, anxious, or stress symptoms.

Result

In the studied population, the majority experienced symptoms of depression (65.1%), anxiety (69.5%), and perceived stress (74.7%). Caregivers of patients with advanced oral cancer were found to have a higher likelihood of experiencing depression (χ2 (1) = 16.76, p < .001) and anxiety related to unemployment (χ2 (1) = 10.12, p = .001) or insufficient earnings (χ2 (1) = 28.63, p < .001). Additionally, participants with no or little formal education (χ2 (1) = 4.63, p = 0.031) and lower income (χ2 (1) = 28.63, p < .0001) were significantly more likely to experience distress compared to those with higher levels of education.

Conclusion

This study highlights the need for comprehensive support systems for family caregivers of oral cancer patients. Educational programs, financial assistance, and mental health services should be tailored to caregivers' specific needs. Early identification and intervention strategies can help mitigate the psychological impact of caregiving. Further research is essential to develop targeted interventions that enhance the well-being of caregivers and improve the quality of life for both patients and caregivers.

## Introduction

Oral cancer is a significant health issue in India. It is the second-most common cancer in India, second-most common amongst men (8.4% of all cancers), and seventh-most frequent among women (3.2% of all cancers) [1,2]. Of the worldwide incidence of cancer, South-Central Asia carries a burden of 11%, of which 1.01% of cases account for oral malignancy [3]. It has one of the highest age-standardized rates (ASR) of incidence of nine per 100,000 people globally [4]. Moreover, the Global Cancer Observatory (GLOBOCAN) has also predicted that if such a trend continues, the number of cancer cases in India will increase to 2.08 million by 2040, accounting for a rise of 57.5% from 2020 [5]. Population-based cancer registries within the National Cancer Registry Programme and outside the network describe a heterogeneous trend of cancers in India. Central India leads in oral cancer representation, making it a pressing public health issue [6,7].

The life trajectory of individuals who have survived oral cancer is marked by challenges during and after treatment. While oral cancer may not be one of the leading global cancers when compared to others such as lung cancer, female breast cancer, prostate cancer, colorectal cancer, stomach cancer, and liver cancer [8], post-treatment physical disfigurement and functional impairment of oral cancer significantly impact the fundamental ability to perform daily tasks and disrupt social functioning, causing difficulties in the daily lives of patients [9-11]. Family caregivers are paramount in improving terminally ill patients' well-being, quality of life, and care [12,13]. Providing care for individuals battling cancer can result in caregiver stress and emotional challenges that intensify as the patient's well-being deteriorates, particularly as they approach the end of life [14,15].

Caregiver stress is an essential predictor for early and recurrent hospitalization of patients. Caregivers caring for patients with diverse medical conditions frequently encounter sleep problems and feelings of depression [16]. Furthermore, on a social level, family caregivers risk facing financial difficulties, social isolation, and work-related challenges while fulfilling their caregiving responsibilities [13,17-21]. Family caregivers of patients in palliative care encounter diverse psychological problems, including high rates of anxiety and depression, reaching up to 40% [13,22]. They often experience distress [22], exhibit symptoms of post-traumatic stress disorder [23], and grapple with emotional issues like helplessness, uncertainty, and hopelessness [14,24]. Furthermore, within the family caregiver population, there is an acknowledgement of adverse outcomes related to bereavement, with around 10-20% of caregivers experiencing complicated grief [25,26]. The psychosocial burden and anxiety experienced by family caregivers of advanced cancer patients are linked to the extent of unmet needs they face [27].

Longitudinal data about the psychological consequences of caring for an advanced cancer family member is limited, and studies investigating interventions in this context are scarce [28]. Despite the significant presence of family caregivers for individuals with advanced diseases within most populations, their needs have received relatively little attention, and their vulnerability appears to have been overlooked. Consequently, there is a need for prospective studies. Therefore, the current study aims to determine the prevalence of anxiety, depressive disorders, and perceived stress among family caregivers of oral cancer patients while examining the associated psychosocial factors.

## Materials and methods

This is an analytical, cross-sectional study conducted at the Clinical Oncology Department (Siddharth Gupta Memorial Cancer Hospital) of Acharya Vinoba Bhave Rural Hospital, Wardha, Maharastra. The hospital serves urban and rural areas, catering to individuals from various social classes. All cases of oral cancer from March 2023 to August 2023 in which consistent caregivers were present, were assessed, along with their respective caregivers. All patients scheduled to visit the outpatient clinic, regardless of being in the postoperative phase or presenting with advanced metastatic disease and awaiting decisions regarding their treatment plan, including chemotherapy or radiotherapy, were invited to partake in the research study.

Participants

A stable caregiver is defined as any person living with the patient, primarily and directly involved in patient care, and affected by the patient's status [16]. The assessment of stable caregivers encompassed various aspects, including their age, gender, relationship to the patient, occupation, monthly income, number of dependents, number of caregivers involved in rotating caregiving duties, other individuals involved in caregiving for the patient, the amount of time dedicated to caregiving, and factors contributing to stress aside from the patient's illness.

Caregivers from a social support network or a professional group were excluded (only two caregivers were excluded). Patients or caregivers who could not complete self-assessment questionnaires were also excluded. An informed consent, which included consent to allow for medical chart data abstraction, was taken from the patients and their caregivers. The study was approved by the Institutional Ethics Committee of Datta Meghe Institute of Higher Education and Research (approval number: DMIHER(DU)/IEC/2023/1307).

Inclusion and exclusion criteria

Caregivers were eligible for the study if they: (i) were identified by the care recipient, who was able to communicate in Marathi/Hindi/English, (ii) provided home care or palliative care no fewer than five days/week or six hours/day, (iii) cared for a patient diagnosed with advanced cancer, (iv) were able to communicate in English, (v) were ≥ 18 years, and (vi) cared for a patient who had a life expectancy of at least one week at the time of study enrolment, as suggested by a Palliative Performance Scale (PPS) [9] score of ≥ 30. The PPS, a modified Karnofsky Performance Scale, is a tool for measuring physical status in palliative care and has predictive validity for a median survival of six days at a score ≥ 30 [10], excluding subjects with rapidly declining health status.

Care recipients were excluded if they had cognitive or physical impairments that made it impossible for them to communicate or complete study instruments and/or did not undersign the consent.

Data collection and tools

Socio-demographic and clinical information, including sex, age, education, employment status, and relationship with the patient, were collected for caregivers and patients. Patients also provided information about their cancer and treatment. The patients' caregivers were subjected to clinical psychiatric assessment using a semi-structured diagnostic interview called Structured Clinical Interview for Diagnostic and Statistical Manual of Mental Disorders, Fifth Edition (DSM-5) (SCID 2). It is considered the standard interview to verify diagnoses in clinical trials and is extensively used in other forms of psychiatric research.

Since the Marathi version of SCID-2 is not available, a translated tool of the same with WHO guidelines was employed [29,30]. In the assessment of cross-language concordance for the translated tool, a significant correlation (p < 0.001) was found in 20 out of the 23 items. Similarly, a significant correlation (p < 0.001) was observed in 21 out of the 23 items in evaluating test-retest reliability. The calculated Cronbach's alpha value was 0.89, indicating a high level of internal consistency. Furthermore, the Spearman-Brown sphericity value for the Marathi version was determined to be 0.79, signifying a robust measure of reliability. Following this, the caregivers individually completed the study questionnaires without being aware of each other's responses. In instances where feasible, the caregiver provided the care recipient's demographic information to minimize any burden on the care recipient during the data collection process. If the patient or caregiver could not complete the questionnaire materials independently, the researcher interviewed the care recipient and primary caregiver to complete the data instrument(s)[31]. The caregivers completed three questionnaires given below.

Beck Depression Inventory (BDI)

A version of the BDI [32], a self-reporting rating scale, which was translated into Marathi by the Depression Anxiety Stress Scale (DASS) team at the King Edward Memorial Hospital and Research Centre, Pune, India [33], was used for measuring the emotional, cognitive, and motivational symptoms of depression. The scale consists of 21 items, each containing four alternatives, scored from 0 to 3. The total scores range from 0 to 63. Higher scores on the scale indicate the greater severity of depression.

Manifest Anxiety Scale (MAS)

The MAS was used to assess the anxiety state. This early instrument, derived from the Minnesota Multiphasic Personality Inventory (MMPI) in which the subject has to answer yes or no, was translated into Marathi [18] and could be understood by everyone who could read simple Marathi. The cross-language concordance test for this showed a significant correlation (p = 0.031) in 30 out of 38 items, and the test-retest reliability had a significant correlation (p < 0.001) in 31 out of 38 items. Cronbach's alpha value was 0.82, indicating good internal consistency.

Perceived Stress Scale (PSS-10)

The study employed the PSS-10 [34], which had undergone validation and translation into Marathi [35], to evaluate the participants' stress levels. The Marathi version of the PSS-10 demonstrated satisfactory reliability and validity (p = 0.021) in eight out of 10 items, and the test-retest reliability had a significant correlation (p < 0.001) in all 10 items. Cronbach's alpha value was 0.88, indicating good internal consistency. This scale encompasses two distinct factors. The first factor comprises queries that capture negative emotions, such as distress, anger, or nervousness, and an individual's perceived inability to manage stress effectively. In contrast, the second factor consists of questions that assess positive emotions and the capacity to cope with stressful situations.

Within the scale's 10 items, participants were prompted to reflect on their recent feelings and thoughts, gauging the extent to which they perceived their current life circumstances as unpredictable, uncontrollable, and fraught with stress. Respondents rated the frequency of these feelings and thoughts over the past month using a five-point Likert scale (ranging from 0 = never to 4 = very often). Higher scores on the scale corresponded to heightened perceived stress levels. Furthermore, the PSS-10 correlated with various psychosocial measures, particularly depression and self-perceived health status.

The criteria to categorize the diagnosis for each study tool are given in Table 1.

**Table 1:** Diagnostic criteria for the study variables of the population.

Data Group	Variable Type	Source of Data	Diagnosis Criteria
Beck Depression Inventory Score (BDIS)	Scale	Questionnaire	No depression: 0-14 Mild depression: 14–19 Moderate depression: 20–28 Severe depression: 29–63
Manifest Anxiety Scale Score (MASS)	Scale	Questionnaire	Normal: 0 -16 Mild anxiety: 17-25 Moderate anxiety: 25-36 Severe anxiety: >36
Perceived Stress Scale Score (PSSS)	Scale	Questionnaire	Low Stress: 0-13 Moderate stress: 14-26 High stress: 27-40

Sample size calculation

The sample size was calculated based on the total number of patients in three months in the outpatient department (OPD). This calculation was done according to a formula by Krejcie and Morgan [36]:

${(\chi ^2*N*P (1-P))/(C^2 (N-1)+\chi ^2*P(1-P))}$

where χ2 is chi-square tabulated value at a 5% level of significance, P is proportion =50%, C2 is confidence interval of one choice i.e., 95%confidence level= 0.05, N is total oral cancer patients in oncology OPD = 110 in three months 

Therefore,

S=3.84 X 110 X 0.5 X 0.5 / ((0.05X0.05X109) + (3.84X0.5X0.5)) 

= 79.349 

~80 samples 

**Figure 1:**
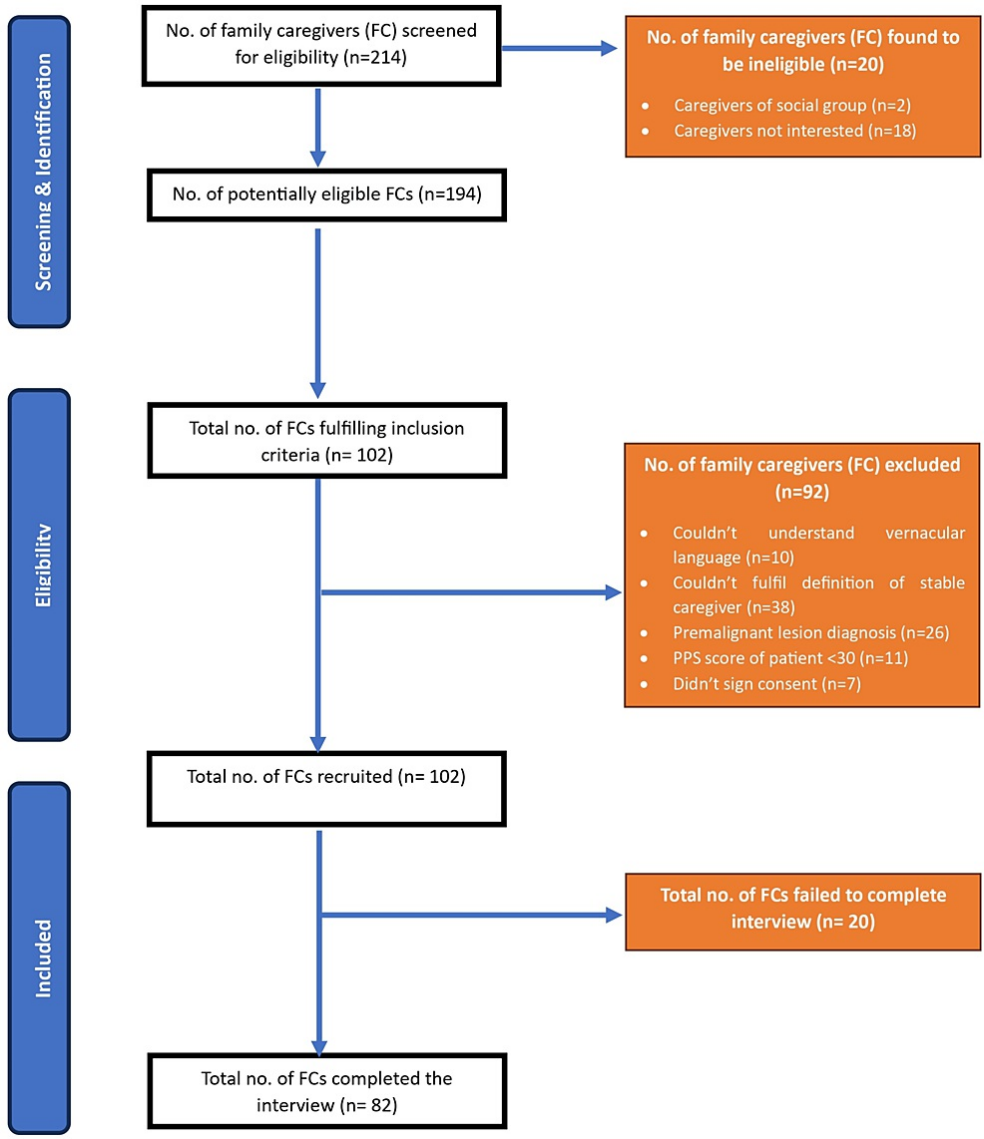
Flowchart of the study protocol as per STROBE guidelines STROBE: Strengthening the Reporting of Observational Studies in Epidemiology

As per the calculations, the sample size was calculated to be 80. The study followed the Strengthening the Reporting of Observational Studies in Epidemiology (STROBE) guidelines and Figure 1 delineates the meticulously structured study protocol, elucidating the stepwise inclusion and exclusion of participants at each juncture, cataloguing the reasons underpinning these decisions. In the end, a total of 82 caregivers were interviewed.

Statistical analysis

Results were analyzed by IBM SPSS Statistics for Windows, Version 26.0 (Released 2019; IBM Corp., Armonk, New York, United States). The chi-squared test (χ2) was used to study the association between qualitative variables. Fisher's exact test was used whenever any of the expected cells were less than five. ANOVA test was used to compare quantitative variables between more than two groups of normally distributed data; Kruskal-Walli's test was used to compare quantitative variables between more than two groups of not normally distributed data. A multivariate logistic regression model was performed to ascertain the effect of each risk factor on the intended outcome. P-value < 0.05 was considered significant.

## Results

The participant's characteristics are shown in Table 2. The final sample was composed of 82 caregivers. Both genders were equally represented in the caregivers' group (43 males (52.4%) and 39 females (47.5%)), whereas the patients were primarily males (n=60, 73.1%). There was a substantial difference in age between caregivers (mean age36.1 years, SD=10.5) and patients (mean age59.8 years, SD=12.4). The average family size in the patient's family was approximately three members. Only five patients (6.10%) were not married, 68 (82.92%) were married, and nine (10.98%) were widowed. A significant proportion of caregivers (n=36, 43.90%) and patients (n=45, 54.87%) were categorized as illiterate. Most caregivers (n=32, 39.02%) and patients (n=36, 43.90%) had received primary or secondary education. However, the representation of individuals with higher education levels was minimal among caregivers (n=14, 17.04%) and almost negligible among patients (n=1, 1.21%). Regarding employment status, a considerable proportion of caregivers (n=43, 52.43%) and patients (n=26, 31.70%) were engaged in manual or seasonal work. Furthermore, the high percentage of retired patients (n=18, 21.96%) suggests that caregiving for elderly patients is a prevalent scenario. Meanwhile, unemployment was notable among caregivers (n=17, 20.73%) and patients (n=23, 28.05%), indicating the potential economic strain on families due to the disease burden. The analysis of income levels reveals that a substantial portion of caregivers (n*=*49, 59.76%) and patients (n=39, 47.56%) reported insufficient income. Half of the study's patients (n=41, 50%) had medical comorbidities. A relatively small percentage of patients (n=12, 14.63%) had a family history of psychiatric disorders. The majority of patients were married (n=68, 82.92%), while a smaller percentage had never married (n=5, 6.10%) or were widowed (n=9, 10.98%). Patients were almost evenly distributed between rural (n=38, 46.34%) and urban (n=44, 53.66%) areas. The distribution of patients across different stages of oral malignancy varied, with the majority falling into stages 4a (n=20, 24.39%) and 4b (n=15, 18.29%).

**Table 2:** Baseline sociodemographic data of the study population

	Caregivers, n (%)	Patients, n (%)
Sex		
Male	43 (52.44)	60 (73.17)
Female	39 (47.56)	22 (26.83)
Age (years), mean ± SD	36.1 ± 10.5	59.8 ± 12.4
Education		
Illiterate	36 (43.90)	45 (54.87)
Primary/secondary	32 (39.02)	36 (43.90)
Higher	14 (17.04)	1 (1.21)
Employment Status		
Manual/seasonal worker	43 (52.43)	26 (31.70)
Full-time job	21 (25.60)	15 (18.29)
Retired	1 (1.21)	18 (21.96)
Unemployed	17 (20.73)	23 (28.05)
Income		
Enough	33 (40.24)	43 (52.44)
Not enough	49 (59.76)	39 (47.56)

As per the data for the study variables according to Table 3, the absence of very severe depression in the sample suggests that while caregivers may experience depressive symptoms, they generally fall within the range of mild to moderate severity. The diagnosis of depression among caregivers revealed a spectrum of severity. A significant portion of caregivers did not exhibit symptoms of depression (*n*=29, 34.9%). However, it was highlighted that a noteworthy proportion of caregivers displayed varying degrees of depressive symptoms, including mild (n=19, 22.9%), moderate (n=23, 27.7%), and severe (n=11, 13.3%) depression. The diagnosis of anxiety among caregivers displayed a similar range of severity. A substantial number of caregivers (n=25) were classified as having very low or normal anxiety levels (which can be seen as a positive mental health indicator). Nevertheless, a considerable portion of caregivers experienced varying degrees of anxiety, including mild (n=28, 34.1%), moderate (n=27, 32.9%), and severe (n=2, 2.4%) anxiety. While severe anxiety was less prevalent than at other levels, it is still a notable concern. Perceived stress levels among caregivers were distributed across different categories. Most caregivers reported experiencing moderate stress (n=38, 45.8%). Additionally, a significant proportion of caregivers reported experiencing high stress (n=23, 27.7%). On the positive side, a notable portion of caregivers reported low stress (n=21, 25.3%).

**Table 3:** Depression, anxiety, and stress in the caregivers

Diagnosis in caregivers	Number of caregivers (%)
Depression	
No depression	29 (34.9)
Mild	19 (22.9)
Moderate	23 (27.7)
Severe	11 (13.3)
Very severe	0
Anxiety	
Very low/normal	25 (30.5)
Mild	28 (34.1)
Moderate	27 (32.9)
Severe	2 (2.4)
Perceived Stress	
Low stress	21 (25.3)
Moderate stress	38 (45.8)
High stress	23 (27.7)

As depicted in Table 4, on account of the relationship between the total number of cases and patients' caregivers with depression concerning patient and disease characteristics, the stage of oral cancer exhibited a statistically significant association with depression in caregivers (χ^2^(1) = 16.756, p < .001). The residence of caregivers, whether rural or urban, displayed a marginally significant association with depression (χ^2^(1)= 2.721, p = 0.099). While not statistically significant at the conventional level, it suggests that there may be a trend worth exploring further.

**Table 4:** Association of depression in caregivers with socioeconomic details and patient characteristics *  showing statistical significance (p<0.05)

	Total (N=82), n (%)	Depression in Caregiver (N= 53), n (%)	Test of significance	p-value
Stage				
Early	25 (30.49)	8 (15.09)	Χ^2^= 16.756	0.00*
Advanced	57 (69.51)	45 (84.91)		
Treatment				
Treatment started	47 (57.31)	28 (52.83)	Χ^2^= 1.233	0.267
Not started	35 (42.69)	25 (47.17)		
Marital Status				
Single	14 (17.07)	11 (20.75)	Χ^2^= 1.435	0.231
Married	68 (82.93)	42 (79.25)		
Residence				
Rural	38 (46.34)	21 (39.62)	Χ^2^= 2.721	0.099
Urban	44 (53.66)	32 (60.38)		
Education				
Illiterate	36 (43.91)	22 (41.51)	Χ^2^= 0.348	0.555
Educated	46 (56.09)	31 (58.49)		
Employment Status				
Employed	64 (78.04)	43 (81.13)	Χ^2^= 0.832	0.362
Unemployed	18 (21.96)	10 (18.87)		
Income				
Enough	33 (40.24)	20 (37.73)	Χ^2^= 0.392	0.531
Not enough	49 (59.76)	33 (62.26)		

Income (χ^2^(1)= 28.633, p < 0.001) as well as employment status (χ^2^(1) = 10.115, p = 0.001) emerged as a significant predictor of caregiver anxiety (Table 5).

**Table 5:** Association of anxiety in caregivers with sociodemographic details and patient disease characteristics * showing statistical significance (p<0.05)

	Total (N=82), n (%)	Anxiety in caregivers (n= 57), n (%)	Test of significance	p-value
Stage				
Early	25 (30.49)	17 (29.82)	Χ^2^= 0.390	0.844
Advanced	57 (69.51)	40 (70.18)		
Treatment				
Treatment Started	47 (57.32)	31 (54.39)	Χ^2^= 0.657	0.418
Not started	35 (42.68)	26 (45.61)		
Marital Status				
Single	14 (17.07)	11 (19.30)	Χ^2^= 0.654	0.419
Married	68 (82.93)	46 (80.70)		
Residence				
Rural	38 (46.34)	25 (43.86)	Χ^2^= 0.463	0.496
Urban	44 (53.66)	32 (56.14)		
Education				
Illiterate	36 (43.90)	24 (42.11)	Χ^2^= 0.245	0.620
Educated	46 (56.10)	33 (57.89)		
Employment Status				
Employed	57 (69.51)	39 (68.42)	Χ^2^= 10.115	0.001*
Unemployed	25 (30.49)	18 (31.58)		
Income				
Enough	33 (40.24)	12 (21.05)	Χ^2^= 28.633	0.000*
Not enough	49 (59.76)	45 (78.95)		

Education emerged as a significant predictor of caregiver distress (χ^2^(1)= 4.628,p = 0.031), as shown in Table 6. Caregivers with lower levels of education were significantly more likely to experience distress than those with higher educational attainment.

**Table 6:** Association of stress in caregivers with socioeconomic details and patient disease characteristics

	Total (N=82), n (%)	Distress in Caregiver (n= 61), n (%)	Test of Significance	p-value
Stage				
Early	25 (30.49)	20 (32.79)	Χ^2^= 0.594	0.441
Advanced	57 (69.51)	41 (67.21)		
Treatment				
Treatment started	47 (57.32)	37 (60.66)	Χ^2^= 1.085	0.298
Not started	35 (42.68)	24 (39.34)		
Marital status				
Single	14 (17.07)	11 (18.03)	Χ^2^= 0.155	0.694
Married	68 (82.93)	50 (81.97)		
Residence				
Rural	38 (46.34)	31 (50.82)	Χ^2^= 1.921	0.166
Urban	44 (53.66)	30 (49.18)		
Education				
Illiterate	36 (43.90)	31 (50.82)	Χ^2^= 4.628	0.031*
Educated	46 (56.10)	30 (49.18)		
Employment Status				
Employed	64 (78.05)	50 (81.97)	Χ^2^= 2.135	0.144
Unemployed	18 (21.95)	11 (18.03)		
Income				
Enough	33 (40.24)	26 (42.62)	Χ^2^= 0.561	0.454
Not enough	49 (59.76)	35 (57.38)		

A logistic regression was performed to ascertain the effects of the stage of the disease of the patient, the status of surgery of the patient, marital status of the patient, residence, occupation of caregiver, education of the caregiver, and income on the likelihood that participants have depression, anxiety, and distress (Table 7). The regression analysis for the depression model was statistically significant (χ^2^(7) = 21.976, p = 0.001), and the non-significant Hosmer-Lemeshow test (χ^2^(8) = 4.838, p > 0.05) indicated that the data fit the model well. The model explained 32.3% (Nagelkerke R^2^) of the variance in depression in caregivers and correctly classified 75.6% of cases. The advanced stage of disease of the patient was 13 times more likely to induce depression in his caregiver (OR=13.898, 95%CI [3.155, 61.222]). Similarly, the regression analysis for the anxiety model was statistically significant (χ^2^(7) = 43.787, p< 0.001), and the non-significant Hosmer-Lemeshow test (χ^2^(8) = 4.134, p> 0.05) indicated that the data fit the model well. The model explained 58.5% (Nagelkerke R^2^) of the variance in anxiety in caregivers and correctly classified 86.6% of cases. The caregiver's income was three times more likely to induce anxiety in him/her (OR=3.269, 95%CI [5.612, 123.192]). The anxiety regression model was statistically significant (χ^2^(7) = 12.985, p=0.01), and the non-significant Hosmer-Lemeshow test (χ^2^(8) = 7.058, p>0.05) indicated that the data fit the model well. The model explained 42.3% (Nagelkerke R^2^) of the variance in depression in caregivers and correctly classified 78.0% of cases. The education status of the caregiver was approximately twice as likely to induce distress in him/her (OR=1.674, 95%CI [1.484, 19.176]). Caregivers with lower education levels are at significantly higher odds of experiencing distress. The rest of the covariates were not significant.

**Table 7:** Logistic regression analysis on factors associated with anxiety, depression, and distress * showing statistical significance (p<0.05)

Variables	B	p-value	Odds Ratio, Exp (B)	95%CI	
				Lower	Upper
Depression					
Stage	2.632	0.001*	13.898	3.155	61.222
Surgery	-0.828	0.235	0.437	0.112	1.713
Marital Status	-0.689	0.371	0.502	0.111	2.275
Residence	0.574	0.299	1.775	0.601	5.242
Occupation	0.099	0.888	1.105	0.276	4.420
Education	0.668	0.250	1.950	0.625	6.080
Income	0.254	0.657	1.289	0.420	3.953
Anxiety					
Stage	1.014	0.251	2.756	0.489	15.547
Surgery	0.781	0.322	2.185	0.465	10.259
Marital Status	0.415	0.684	1.514	0.205	11.157
Residence	0.107	0.879	1.113	0.279	4.444
Occupation	21.242	0.998	1679324757.017	0.000	.
Education	0.643	0.357	1.902	0.485	7.461
Income	3.269	0.000*	26.295	5.612	123.192
Distress					
Stage	0.857	0.273	2.356	0.508	10.924
Surgery	0.323	0.624	1.382	0.379	5.032
Marital Status	0.710	0.369	2.033	0.432	9.569
Residence	0.505	0.386	1.657	0.529	5.196
Occupation	1.307	0.081	3.694	0.853	16.006
Education	1.674	0.010*	5.335	1.484	19.176
Income	0.181	0.765	1.199	0.365	3.935

## Discussion

The results divulged in the findings section illuminate several pivotal facets of caregiver mental well-being concerning their role in providing care to patients afflicted with oral malignancies. Within the examined population, a substantial majority exhibited indications of depressive symptoms (65.1%), anxiety (69.5%), and perceived stress (74.7%). This aligns with the observations made by Hui et al. [37] and Badr et al. [38]; both underscore the heightened prevalence of psychological distress among caregivers of oral cancer patients. Furthermore, their research underscores that caregivers are disproportionately susceptible to experiencing severe manifestations of anxiety and depression.

The outcomes of the current investigation exhibited higher prevalence rates than the study conducted by Gondivkar et al. [39], which reported anxiety prevalence ranging from 40% to 50% among the sampled oral cancer patients' caregivers. This heightened psychological distress can be contextualized within India's rapid urbanization and the prevailing trend toward nuclear family structures. Caring for relatives residing in rural areas has become a notably burdensome task. As a result, family caregivers of individuals grappling with advanced stages of cancer find themselves trapped with a substantial psychological burden that commences with their relative's initial cancer diagnosis and persists well beyond the bereavement phase.

Upon evaluating the severity of depression and anxiety disorders, it was found that most cases were classified as moderate to severe anxiety (69.5%) and depression (65.1%). Severe psychiatric symptoms were uncommon among caregivers of early-stage oral cancer patients with good prognoses but more prevalent among patients with advanced stages.

This study rigorously examined the occupational status of caregivers to ascertain any potential links with psychiatric morbidity. The results revealed that a substantial proportion of patients engaged in seasonal or manual work, accounting for 52.4% of the sample, and 59.7% grappled with inadequate monthly income. This situation pointedly indicated a modest standard of living, primarily attributable to the elevated cost of living in an urban setting. Importantly, it should be noted that certain patients resided in rural areas where expenses related to transportation and accommodation could be notably burdensome. Consequently, an overwhelming majority of patients unequivocally conveyed their distress concerning the financial aspects of cancer treatment, particularly regarding their economic status.

The study's findings revealed a notable prevalence of distress within the study group, amounting to 74.4%. These results align with the research conducted by Ross et al. [40], which indicated that 50% of their subjects experienced high distress. Additionally, studies by Zabora et al. [41] and Fallowfield et al. [42] have consistently reported the prevalence of psychological distress in cancer patients to be above 30%.

Furthermore, educational attainment emerged as a pivotal factor influencing caregiver distress and anxiety. Notably, individuals with lower levels of education exhibited a significantly higher likelihood of experiencing distress [43]. This observation underscores the critical need for educational initiatives tailored to caregivers, particularly those with limited formal education [44].

Caregiver characteristics, including gender distribution and age, reflect a diverse cohort in the current study, and the mean age of the caregiver group was 36.12 ± 10.52 years. This was consistent again with Gondivkar [39], who found that most patients were in the age group of 28-52 years followed by the age group of 30-58 years. The mean age of the patients was 59.77 ± 12.36 years. This finding aligned with the National Cancer Registry 2020 [6]. The substantial age gap between caregivers and patients, with caregivers being significantly younger, underscores the generational dynamics inherent in caregiving for individuals with oral malignancies. Providing care for elderly individuals extends its purview across the entire spectrum of care-delivery contexts.

Close to three-quarters of the patients were male (73.2%), the majority were married (82.9%), and most were illiterate (54.9%). This finding was also supported by the study conducted by Unsar et al. [45]. Notably, the caregiver group in this study was marginally dominated by married males, similar to the study by Kondeti et al. [46]. On the contrary, according to the study done by Schrank et al. [47], women were found to bear a significant burden of caregiving in oral cancer patients. Similarly, Goswami et al.'s study in the area backs up the dissimilar findings [48]. In an international setting, a Brazilian study conducted by Rigoni et al. [49] found that 76.7% of the caregivers were women. Röing et al. suggested that the emotional bond between the caretaker and patient if the caretaker was the wife, could affect the study results by making patients more prone to emotional changes [50].

In contrast to the study conducted by Govina et al. [51], concerning the burden experienced by family members caring for patients with advanced cancer, the patient population in our study was predominantly male. This observation highlights oral malignancies' gender-specific burden, primarily affecting males. Pinquart et al. reported that compared to grown-up children serving as caregivers, spouses tended to report worse physical health and were more susceptible to physical decline due to age-related factors [52]. However, it's worth noting that Ross et al. argue that the caregiver's gender did not significantly impact the psychosocial well-being of the caregiver [40].

The diagnosis of cancer within a family significantly influences the dynamics of relationships and roles among family members, who play pivotal roles in the caregiving process [53-55]. Compelled by the bonds of kinship and constrained by financial limitations, family caregivers often shoulder the exclusive responsibility for caring for their ailing relatives. This predicament is further exacerbated by the limited availability and exorbitant costs associated with formal caregiving services, particularly pronounced in South Asian countries like India [55-57]. Consequently, Indian family caregivers confront a dual challenge, encompassing both the daily exigencies of livelihood and the formidable task of providing care to their ailing family members [58-66].

A primary contribution of this cross-sectional investigation lies in its expansion of previous research, effectively addressing a knowledge gap regarding the prevalence of depression, anxiety, and perceived stress among caregivers of oral cancer patients and their associations with socio-demographic variables. The presence of moderate to very severe scores in these domains among family caregivers underscored robust positive correlations with the patient's cancer stage, the caregiver's income level, and the caregiver's educational attainment.

Given these findings, we recommend pursuing future prospective studies with larger sample sizes. Such endeavours could significantly contribute to augmenting healthcare professionals' awareness of the mental healthcare needs of family caregivers, particularly in the initial phases of the illness. This heightened awareness can pave the way for early interventions to arrest the progression or mitigate the symptoms of these psychological conditions.

Limitations of the study

Some limitations are inherent to this study. Firstly, the utilization of a relatively small sample size, primarily drawn from a single healthcare facility with outpatient participants, hinders the broad generalization of our findings. To enhance the robustness and applicability of these results, future research endeavours should consider conducting longitudinal investigations on more extensive and diverse population cohorts. The study's generalizability could be bolstered by expanding participant recruitment to encompass individuals from diverse settings and regions across the country. Moreover, it is imperative to acknowledge that our study did not account for the non-response rate in caregiver interviews, which could introduce selection bias. Furthermore, the current analysis did not include certain pertinent factors contributing to caregiver distress, such as pain levels, availability of financial support, family background, physical suffering, and social support. Addressing these factors in future studies could provide a more comprehensive understanding of the complexities surrounding caregiver well-being. Lastly, our analysis of factors related to the study was confined to data collected within a short three-month timeframe, providing potential for further investigation into its long-term effects.

## Conclusions

The present study delved into the complex landscape of caregiver mental health in the context of patients diagnosed with oral cancer. The findings underscore the necessity for comprehensive support systems, including educational programs, financial assistance, and mental health services, to enhance the well-being of caregivers and optimize their capacity to provide adequate care.

Further research in this domain is warranted to develop targeted interventions that address the specific needs of caregivers, often unnoticed by the healthcare team and government in the context of oral cancer and any malignancy, ultimately improving the quality of life for patients and their dedicated caregivers.
